# Evolution of facial identification techniques: A bibliometric analysis (1970 - 2022)

**DOI:** 10.1007/s12024-026-01307-5

**Published:** 2026-07-24

**Authors:** Luiz Alberto Marques, Annie Seabra Medeiros Antunes, Thaiza Gonçalves Rocha, Carla Barros de Oliveira, Márcia Barcelos Gomes Silvares, José Vicente-Gomila, Mariana Leonel Martins, Lucianne Cople Maia, Andréa Fonseca-Gonçalves, Andreia Cristina Breda de Souza, Maria Augusta Visconti

**Affiliations:** 1 Postgraduate Program, Professional Masters Degree in Clinical Dentistry, Universidade Federal do Rio de Janeiro, Rua Professor Rodolpho Paulo Rocco, 325 – Cidade Universitária, Rio de Janeiro, CEP: 21941-971 RJ Brazil; 2 Department of Pathology and Oral Diagnosis, School of Dentistry, Universidade Federal do Rio de Janeiro, Rua Professor Rodolpho Paulo Rocco, 325 – Cidade Universitária, Rio de Janeiro, CEP: 21941-971 RJ Brazil; 3 Central Library of the Health Science Center, Universidade Federal do Rio de Janeiro, UFRJ - Ilha do Fundão, Avenida Carlos Chagas Filho, Bl L, 373 - Cidade Universitária, 21941-902 Rio de Janeiro - RJ, Brazil; 4 Department of Enterprise Management, E.S.I.C. Business & Marketing School, Av. de Juan XXIII, 12, Espanha 28224 Pozuelo de Alarcón, Madrid, Spain; 5 Department of Pediatric Dentistry and Orthodontics, School of Dentistry, Universidade Federal do Rio de Janeiro, Rua Professor Rodolpho Paulo Rocco, 325 – Cidade Universitária, Rio de Janeiro, CEP: 21941-971 RJ Brazil; 6 Department of Preventive and Social Dentistry, School of Dentistry, Universidade Federal do Rio de Janeiro, Rua Professor Rodolpho Paulo Rocco, 325 – Cidade Universitária, Rio de Janeiro, 21941-971 RJ Brazil

**Keywords:** Forensic sciences, Face, Photography, Facial recognition, Anthropometry, Forensic imaging

## Abstract

Purpose. This study performed a bibliometric analysis of global research on human facial recognition for forensic purposes, identifying trends in techniques, anatomical regions, and methodological evolution over five decades. Methods. Six databases (PubMed, Scopus, Web of Science, Embase, BVS, and Lilacs) were searched without language or year restrictions. We included studies focusing on human identification through clinical or imaging facial examinations. Data regarding year, journal, authors, keywords, age group, examination type, and methods (metric, morphological, or volumetric) were extracted. Two independent investigators used Rayyan™ for selection, and descriptive analysis was performed using VantagePoint™ and Excel™. Results. From 5016 identified articles, 424 studies published between 1970 and 2022 were included (four systematic reviews, three clinical, 377 observational, and 40 laboratory studies). Forensic Science International was the leading journal (*n* = 113), and Stephan CN was the most prolific author. Photography was the most used examination (*n* = 125). Metric analysis prevailed as the primary method (*n* = 221). While the total face was the most frequent focus (*n* = 239), the nose emerged as the most studied isolated region (*n* = 20). Most studies (*n* = 415) reported identification results. Conclusion. Facial identification research has grown significantly, with photography and metric methods remaining the current standards. However, bibliometric trends point toward a paradigm shift: the integration of Artificial Intelligence and 3D volumetric analysis is emerging as a critical frontier to overcome the limitations of conventional 2D techniques, offering superior precision and optimized forensic workflows.

**Supplementary Information:**

The online version contains supplementary material available at https://doi.org/10.1007/s12024-026-01307-5.

## Key points

Most studies analyzed the entire face and confirmed identification.

Photography and metric analysis were the most widely used techniques; however, volumetric analyses are becoming more prominent.

Digital methods and the use of anthropometric points prevail as the main techniques, pointing to the increasing integration of artificial intelligence in diagnostic imaging protocols.

**Supplementary Information:**

The online version contains supplementary material available at https://doi.org/10.1007/s12024-026-01307-5.

## Introduction

Post-mortem (PM) human identification is essential in society, making the area of Legal Medicine and Forensic Sciences an activity with a fundamental role in legal, social, and humanitarian aspects. No less important for society is the role of forensic experts in human identification in life, often directly linked to criminal expertise [1, 2]. The need to identify an individual converges with the obligations established by law, in addition to meeting the fundamental right of the citizen to have an identity, both in life and after death, also respecting the right of families to define the destiny of relatives, in case of death [3].

The International Criminal Police Organization (INTERPOL) classifies fingerprint analysis, genetic recognition (DNA profiling), and Forensic Dentistry as primary methods of human identification [4]. However, there are intrinsic biological factors and characteristics acquired by people that add information to countless other forensic identification techniques and methods, such as body odor, vein morphology, hand geometry, tattoos, gait type, body morphology, healed bone fractures, soft tissue scars, nevi and freckles, facial analysis, morphology and volumes of the sinuses and nasal septum, iris, or retina.

Emphasis should be given to human identification through the face, either directly or through images, since recent studies [3, 5] that evaluated the main methods of human identification concluded that in 68% to 91% of the cases evaluated, the individuals were identified through visual methods of facial identification. This identification work is highly delicate and complex. It is usually carried out by a multidisciplinary team of specialists [3, 6], where records such as radiographic images (medical/dental), computer tomography (CT) scans, facial scans, ultrasounds, and photographs constitute essential material to be investigated and analyzed [1, 7, 8].

Thus, human identification methods that use facial images are of great value and indispensable in many circumstances, providing routine means of police investigation and valuable evidence in unsolvable cases [9]. In addition, many identification techniques, such as forensic facial approximation [10], photographic overlay, photomontage, facial representation, and facial comparison, use these images as a resource, bringing enormous contributions to Forensic Anthropology by exponentially increasing access to detailed information about an individual to be identified [11].

On the other hand, when there are no previous records that can help assertively identify the individual, a probable identification is achieved by descriptions of physical aspects, including the facial features. Therefore, all evidence must be consistent and noted. The greater the number of descriptions, the more reliable the process will be [12]. Considering that the union between human beings and imaging technology guarantees high precision in human identification through the face [13] and given the variety of techniques and methods available for facial recognition, a bibliometric analysis of the studies related to human recognition through the face becomes necessary to provide the forensic specialist with all the dynamics and evolution of the techniques used worldwide over the years.

## Materials and methods

### Information sources and search strategy

Although there is no specific PRISMA (Preferred Reporting Items for Systematic Reviews and Meta-Analyses) checklist for bibliometric analyses, the authors ensured the transparency of the process by following the applicable elements of PRISMA 2020 for the selection and provided exhaustive details of the search strategy.

A search was carried out by one researcher (A) guided by an expert librarian in December/2022 with no restriction on date or language. The electronic databases searched were PubMed, Scopus, Web of Science, Embase, and LILACS (via V.H.L.), to select scientific articles that reported using the face in human recognition. MeSH terms, entries, accessible terms, and Boolean operators (OR and AND) were used, adapted according to the syntax rules of each electronic database mentioned above (Table 1), and organized according to the search strategy considering studies with individuals in any age group that were exposed to face exams to human identification. This review was registered in the Open Science Framework (OSF) https://osf.io/3wjqa.

**Table 1:** Search strategy for each database

Electronic databases	Search strategy
PubMed https://pubmed.ncbi.nlm.nih.gov/pubmed	(Facial Recognition[MeSH] OR “Facial Recognition“[TiAb] OR Recognition Facial[TiAb] OR “Face Recognition“[TiAb] OR “Recognition Face“[TiAb] OR Biometric Identification[MeSH] OR Biometric Identification[TiAb] OR Facial Identity Recognition[TiAb] OR Identity Recognition Facial[TiAb] OR Recognition Facial Identity[TiAb] OR Human Identification*[TiAb] OR Identification* Human[TiAb] OR Face[MeSH] OR Face[TiAb] OR Automated Facial Recognition[MeSH] OR Automated Facial Recognition[TiAb] OR Faces[TiAb] OR Face Processing[TiAb]) AND (Forensic Sciences[MeSH] OR “Forensic Sciences“[TiAb] OR Forensic Anthropology[MeSH] OR “Forensic Anthropology“[TiAb] OR Anthropometry[MeSH] OR Anthropometry*[TiAb] OR “Anthropology Forensic“[TiAb] OR Legal Medicine[TiAb] OR “Forensic Odontology“[TiAb] OR Forensic Medicine[MeSH] OR “Forensic Medicine“[TiAb] OR Forensic Dentistry[MeSH] OR “Forensic Dentistry“[TiAb]) AND (Facial Tomography[All Fields] OR Photography[MeSH] OR Facial Photography[All Fields] OR Cone-Beam Computed Tomography[MeSH] OR Cone-Beam Computed Tomography[TiAb])
Scopus https://www.scopus.com/home.uri	TITLE-ABS-KEY ( “Facial Recognition” OR “Recognition Facial” OR “Face Recognition” OR “Recognition Face” OR “Biometric Identification” OR “Facial Identity Recognition” OR “Identity Recognition Facial” OR “Recognition Facial Identity” OR human AND identification* OR “Identification Human” OR face* OR “Automated Facial Recognition” OR “Face Processing” ) ) AND ( TITLE-ABS-KEY ( “Forensic Sciences” OR “Forensic Anthropology” OR anthropometry OR anthropometry* OR “Anthropology Forensic” OR “Legal Medicine” OR “Forensic Odontology” OR “Forensic Medicine” OR “Forensic Dentistry” ) ) AND ( TITLE-ABS-KEY ( “Facial Tomography” OR photography OR“Cone-Beam Computed Tomography” ) )
Web of Science www.webofscience.com	(“Facial Recognition” OR “Recognition Facial” OR “Face Recognition” OR “Recognition Face” OR “Biometric Identification” OR “Facial Identity Recognition” OR “Identity Recognition Facial” OR “Recognition Facial Identity” OR Human Identification* OR “Identification Human” OR Face* OR “Automated Facial Recognition” OR “Face Processing” ) (Topic) and (“Forensic Sciences” OR “Forensic Anthropology” OR Anthropometry OR Anthropometry* OR “Anthropology Forensic” OR “Legal Medicine” OR “Forensic Odontology” OR “Forensic Medicine” OR “Forensic Dentistry”) (Topic) and (“Facial Tomography” OR Photography OR “Cone-Beam Computed Tomography”) (Topic)
Embase https://www.embase.com	(‘recognition facial’ OR ‘face recognition’/exp OR ‘face recognition’ OR ‘recognition face’ OR ‘biometry’/exp OR biometry OR ‘biometric identification’/exp OR ‘biometric identification’ OR ‘facial identity recognition’ OR ‘identity recognition facial’ OR ‘recognition facial identity’ OR ‘human identification’ OR ‘identification human’ OR ‘face’/exp OR face OR ‘facial recognition’/exp OR ‘facial recognition’ OR ‘face processing’/exp OR ‘face processing’ AND ‘recognition facial’ OR ‘face recognition’/exp OR ‘face recognition’ OR ‘recognition face’ OR ‘biometry’/exp OR biometry OR ‘biometric identification’/exp OR ‘biometric identification’ OR ‘facial identity recognition’ OR ‘identity recognition facial’ OR ‘recognition facial identity’ OR ‘human identification’ OR ‘identification human’ OR ‘face’/exp OR face OR ‘facial recognition’/exp OR ‘facial recognition’ OR ‘face processing’/exp OR ‘face processing’ AND ‘facial tomography’ OR ‘photography’/exp OR photography OR ‘facial photography’ OR ‘cone beam computed tomography’/exp OR ‘cone beam computed tomography’)
BVS and LILACS https://pesquisa.bvsalud.org/portal/	(mh:”Facial Recognition” OR mh:”Reconhecimento Facial” OR tw:”Facial Recognition” OR tw:”Reconhecimento Facial” OR tw:”Recognition Facial” OR tw:”Face Recognition” OR tw:”Recognition Face” OR mh:”Biometric Identification” OR mh:”Identificação Biométrica” OR tw:”Biometric Identification” OR tw:”Identificação Biométrica”) (mh:”Forensic Sciences” OR mh:”Ciências Forenses” OR tw:”Forensic Sciences” OR tw:”Ciências Forenses” OR mh:”Forensic Anthropology” OR mh:”Antropologia Forense” OR tw:”Forensic Anthropology” OR tw:”Antropologia Forense” OR mh: Anthropometry OR mh: Antropometria OR tw: Anthropometry OR tw: Antropometria OR mh:”Forensic Medicine” OR mh:”Medicina Legal” OR tw:”Forensic Medicine” OR mh:”Forensic Dentistry” OR mh:”Odontologia Legal” OR tw:”Forensic Dentistry” OR tw:”Odontologia Legal”) (mh: Photography OR mh: Fotografação OR tw: Photography OR tw: Fotografia OR tw: Facial Tomography OR mh:”Cone-Beam Computed Tomography” OR mh:”Tomografia Computadorizada de Feixe Cônico” OR tw:”Cone-Beam Computed Tomography” OR tw:”Tomografia Computadorizada de Feixe Cônico”)

### Selection of studies and eligibility criteria

The studies retrieved from the electronic databases were exported to Rayyan™ to manage the retrieved references. Firstly, the duplicates were eliminated. Subsequently, two blind and independent researchers (A and B) selected the studies by reading the title and abstract. The full article was read if insufficient data in these sections were observed. If, for some reason, the article was unavailable, an attempt to contact the authors through e-mail messages was performed. If contact was not established after three attempts, the article was excluded. Clinical, laboratory, observational studies, and systematic reviews that used the face as a tool for human recognition were included. Thus, the faces could be evaluated through clinical examinations or by images. Studies that used morphological, volumetric, and metric analyses and manual, digital, or both (manual + digital) analysis methods were included. The exclusion criteria adopted were study designs such as case reports or series of cases, non-systematic reviews, books, book chapters, letters to the editor, editorial, recommendations, errata, and clinical trial protocols, as well as studies that analyzed the iris, teeth and other intraoral structures and facial sinuses.

The conflicts that arose during the selection of studies by the two independent researchers were resolved in a consensus meeting. If any conflict persisted, it was judged by a third experienced and expert researcher in forensic dentistry (C).

### Data extraction about the bibliometric variables and study characteristics

The included studies were transferred to Microsoft Excel™ to perform the studies’ categorization. One researcher (A) manually extracted data from these studies, while another researcher (D) checked the collected data. For the bibliometric analyses, the following variables were considered: year of publication of the scientific article, journal, authors, and keywords. The considered recovery rate criteria were above 95%. The data from authors and journals were disambiguated by three researchers (A, D, and E). The lists the variables collected and their categorical definitions are described in Table 2.

**Table 2:** Variables collected and their categorical definitions

#	Variable	Categorical Definitions and Coding Rules
1	Age group	Classified into: Child (0–10 years); Adolescent (11–19 years, following WHO standards); Adult (20–59 years); and Elderly (≥ 60 years). Studies not specifying ages or involving cadavers were marked as "Not specified" or "Not applicable”.
2	Type of study	Classified as: Clinical, Laboratory, Observational, or Systematic review.
3	Type of examination	Includes: Photography, Facial scanning, Video recording, Computed tomography, Radiography, Magnetic resonance, Ultrasound, or Visual/Clinical examination. If two or more were used, it was coded as "More than one type”.
4	Method of analysis	Manual: Exclusively direct physical measurements using instruments like calipers or protractors on the subject/object. Digital: Any computer-based interaction, including software-assisted landmarking on screen, automated systems (AFRS), or 3D digital processing. Both: Simultaneous use of physical and digital tools.
5	Type of analysis	Metric: Quantitative comparisons of sizes, indices, or distances. Morphological: Qualitative assessment of shapes and facial features. Volumetric: 3D analysis of space/volume. Note: In cases of overlap, studies were classified by their primary reported outcome.
6	Face regions	Single: Only one region studied (e.g., eyes, ears, lips, or nose). More than one: Between two and four facial regions studied simultaneously. Total face: Studies evaluating the facial block as a whole or more than four combined regions, as reported in holistic identification studies.
7	Results on recognition	Positive: Methods that presented satisfactory effectiveness for human recognition according to the authors’ findings. Negative: Methods that failed to provide reliable identification or satisfactory results. Note: These labels reflect qualitative trends and effectiveness, not a quantitative success rate.

### Data analysis

All analyses were performed in VantagePoint^™^ and Microsoft Excel^™^. For bibliometric evaluation, two types of analysis were performed: (1) the autocorrelation between principal authors (with six or more studies published) that was represented by a map, showing the collaboration (co-publications) among them; and (2) the co-occurrence between the year of publication and the leading journals (with four or more studies published), and between the prominent authors and the leading journals. The co-occurrence analyses were represented by bar and column charts, respectively.

Descriptive analyses were performed to demonstrate the type of study and other variables described above. A word cloud with the main keywords was created based on those cited by 20 or more studies, emphasizing that the frequency of citations is proportional to the word size in this type of figure. The propensity of exam types (from 1970 to 2022) was represented through a bubble chart, where the more significant the circle, the greater the numerical values of the investigated issue. Also, other bubble charts indicated the correlation between the type of analyses performed and the regions investigated.

The co-occurrence between the results of the selected studies and the face regions analyzed was performed through a matrix viewer. Subsequentially, column charts represented correlations between the type of analysis and methods and between the age group and the type of exams. A pivot plot was created and exemplified by a table to demonstrate the correlation between three variables: type of exam, face regions, and type of analysis performed.

## Results

**Fig. 1:**
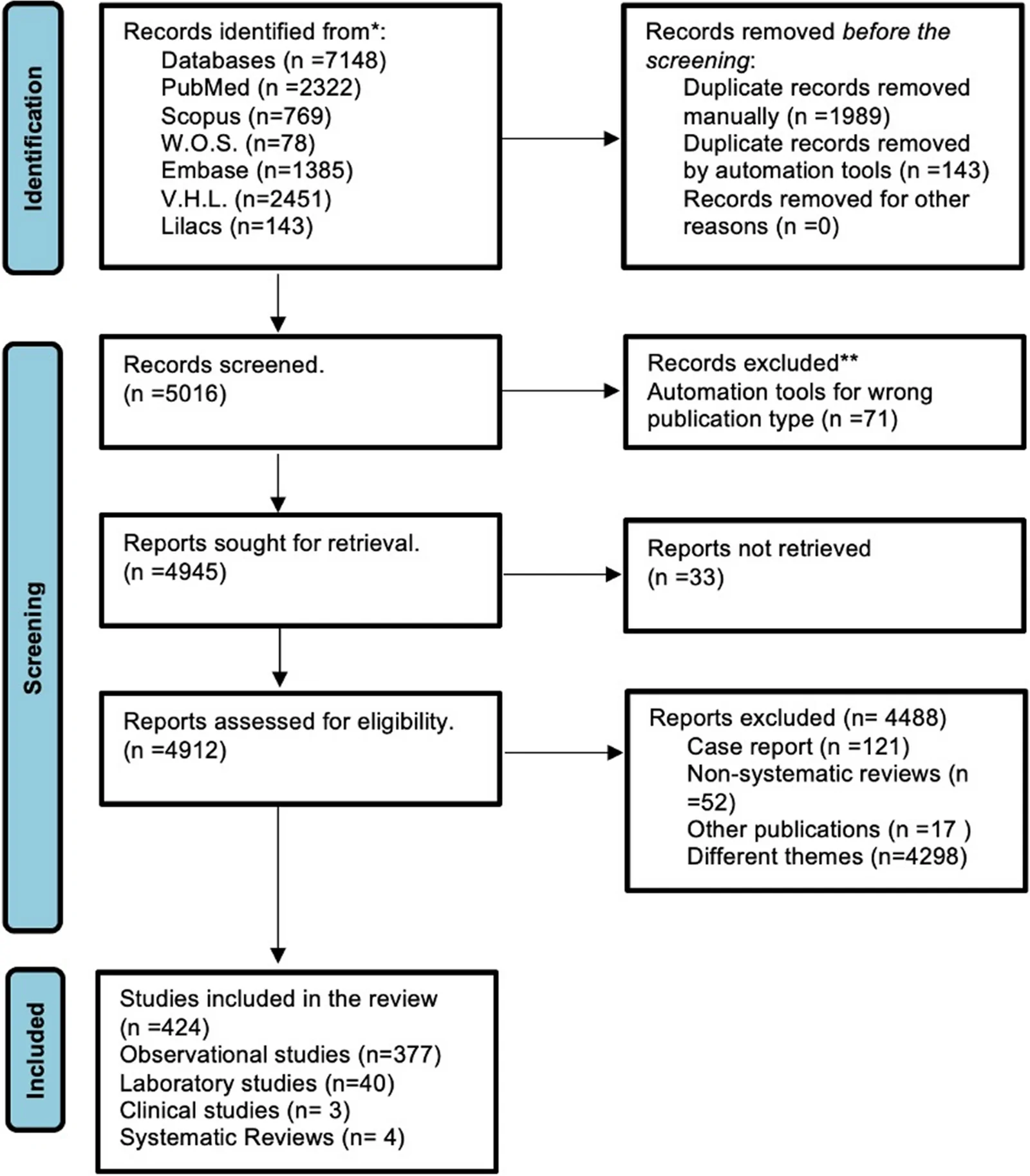
PRISMA flow diagram of the search results from databases

A total of 7148 articles were found after searching the databases. From this total, 2132 were excluded after the duplicate removal, with 1989 records marked as possible duplicates by the Rayyan™ manager and confirmed by a researcher (A) and 143 removed by software automation tools as accurate duplicates. Of the 5016 records, 71 were excluded by the automation tool of the manager’s exclusion criteria, identifying types of incorrect publications such as letters, book chapters, editorials, recommendations, and errata. After searching the remaining 4945 for retrieval, 33 records were not retrieved for reading. The 4912 remaining were submitted to title and abstract screening, and 4488 did not meet the inclusion criteria and were excluded due to the reasons reported in the flow chart (Fig. 1). Finally, 424 articles were included in the present review: 377 observational studies, 40 laboratory studies, three clinical studies and four systematic reviews. Moreover, the studies were published over a period of 53 years, between 1970 and 2022. The period between 2011 and 2022 was the highest number of publications (*n* = 283), corresponding to 66.75% of all the included studies.

### Bibliometric data

**Fig. 2:**
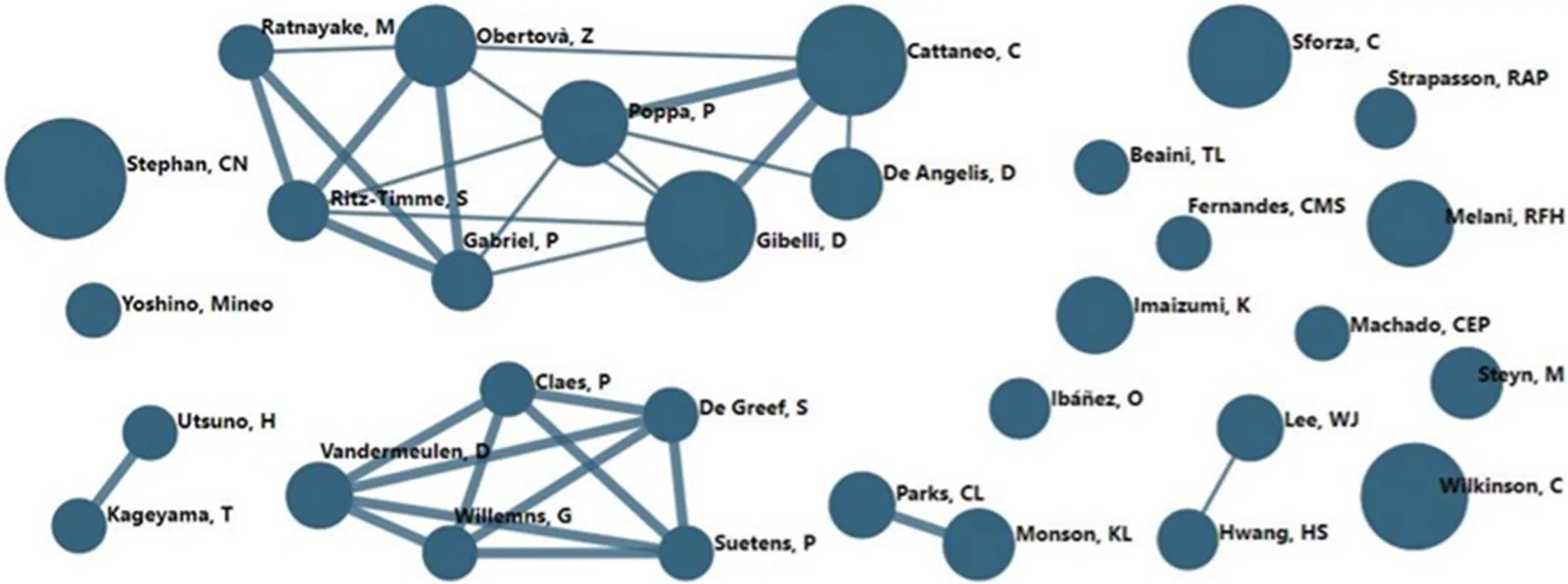
Autocorrelation map between the main authors (six or more published studies). * If a node is alone, it shows no collaboration with the authors on the map. Only strong correlations are shown in the map, represented by thicker lines

Among the 31 prominent authors, Stephan C.N. (21 publications), followed by Cattaneo C. and Gibelli B. (both with 18 publications), and Wilkinson C.M. (17 publications), presented outstanding productivity with the highest number of publications. A broader analysis of collaboration among the authors with six or more publications on the topic showed the existence of two main collaboration clusters, as seen in Fig. 2.

**Fig. 3:**
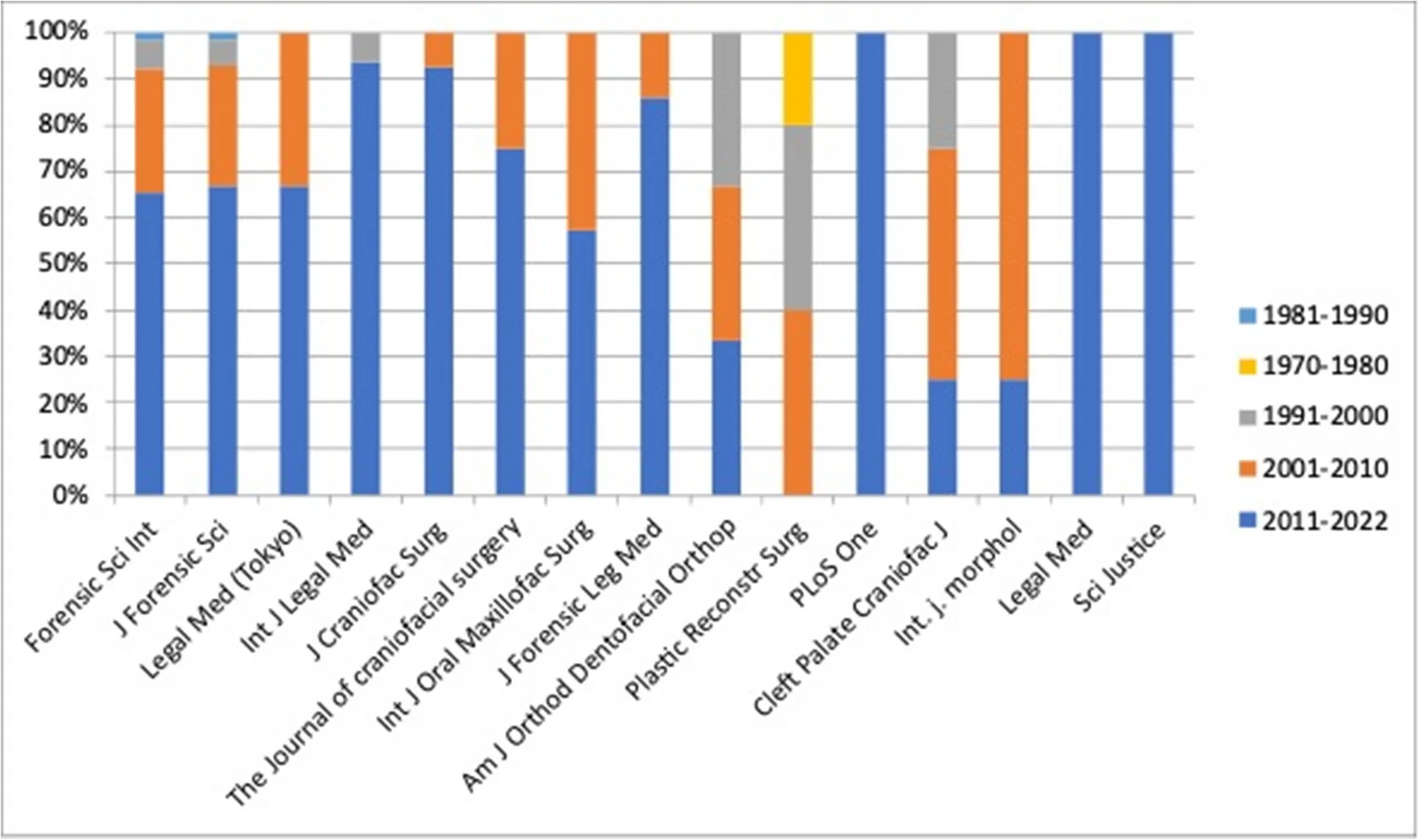
Leading journals that published studies about human identification through the face over the years

In selecting studies that used the face as a method of human recognition, 12 journals with the highest number of publications about this issue stand out. Forensic Science International (*n* = 113–26.66%), Journal of Forensic Sciences (*n* = 56–13.21%), and Legal Medicine Journal - Tokyo (*n* = 15–3.54%) ranked as the first three. The Legal Medicine Journal (*n* = 4–0.95%), PLOS ONE (*n* = 5–1.18%), and Science & Justice (*n* = 5–1.18%) had 100% of the publications included in the period between 2011 and 2022. Only one study among the journals that published four or more articles was published in the period between 1970 and 1980 (Fig. 3).

**Fig. 4:**
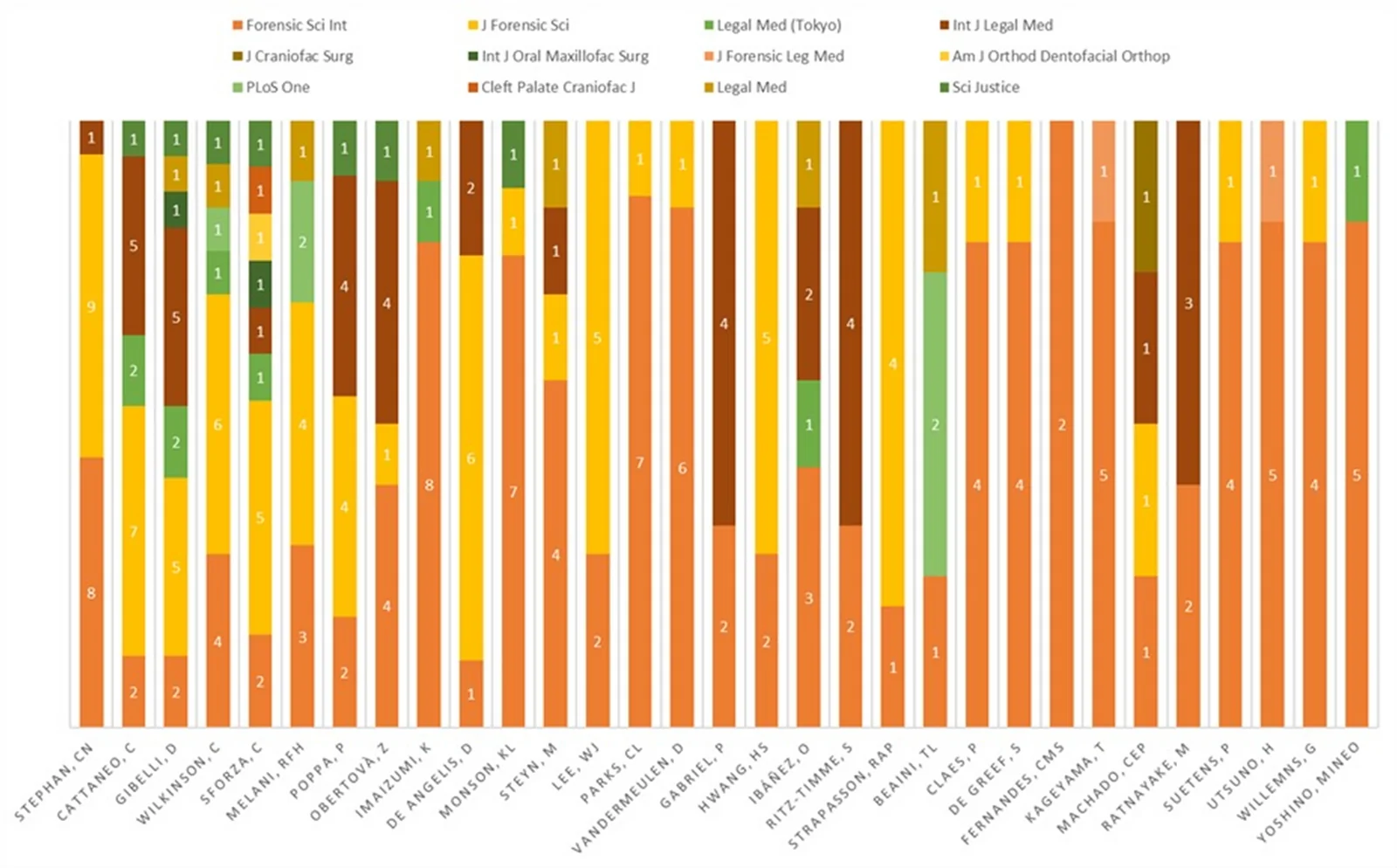
Distribution of the principal authors and the leading journals in which they published their studies

Considering the leading journals, it was observed that a third of the primary authors (*n* = 10) published in four or more of them. Sforza C. was the author who published in a more significant number of these journals (*n* = 8). On the other hand, Fernandes C.M.S., with six studies, was published in only one journal (Fig. 4).

**Fig. 5:**
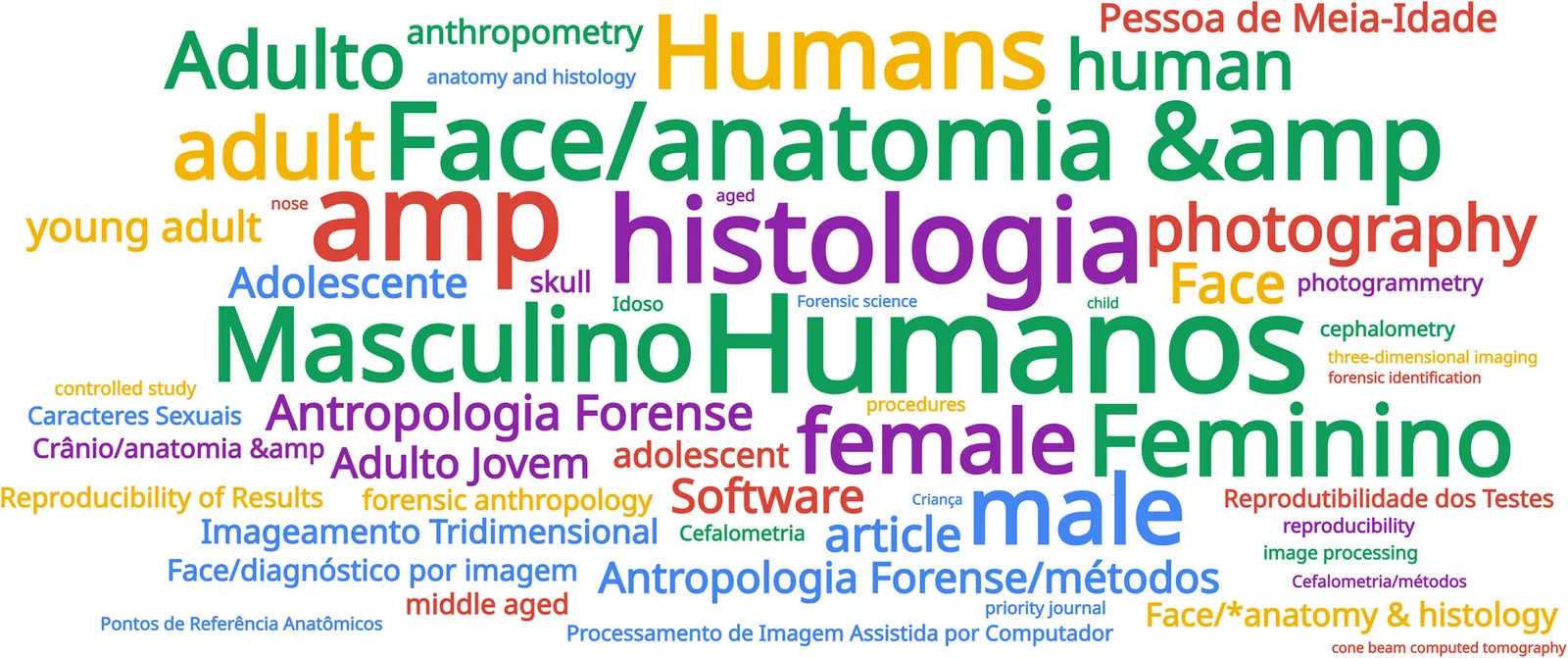
Word Cloud with the keywords cited by 20 or more studies. Note: The term ‘amp’ is a metadata processing artifact representing the ‘&’ symbol and was not considered for scientific analysis

The most common words were “humans,” “histology,” “face/anatomy,” and “male.” “Children” was the word that appeared less frequently. Among the most frequent keywords, terms related to imaging exams (photography) prevailed. Technical artefacts arising from metadata indexing (such as ‘amp’) were identified and excluded from the qualitative analysis (Fig. 5).

## Data related to study characteristics

The isolated exam most used in the selected studies was photography (*n* = 125–29.48%), significantly when associated with the total face region (*n* = 75–17.69%) and when the metric analysis was performed (*n* = 64–15.1%). However, there was a predominance of studies that used more than one type of exam (*n* = 139–32.78%), associated with the study of the total face (*n* = 100) and when several analyses were performed (*n* = 67–15.80%). The last type of exam was magnetic resonance imaging (*n* = 6–1.41%).

**Fig. 6:**
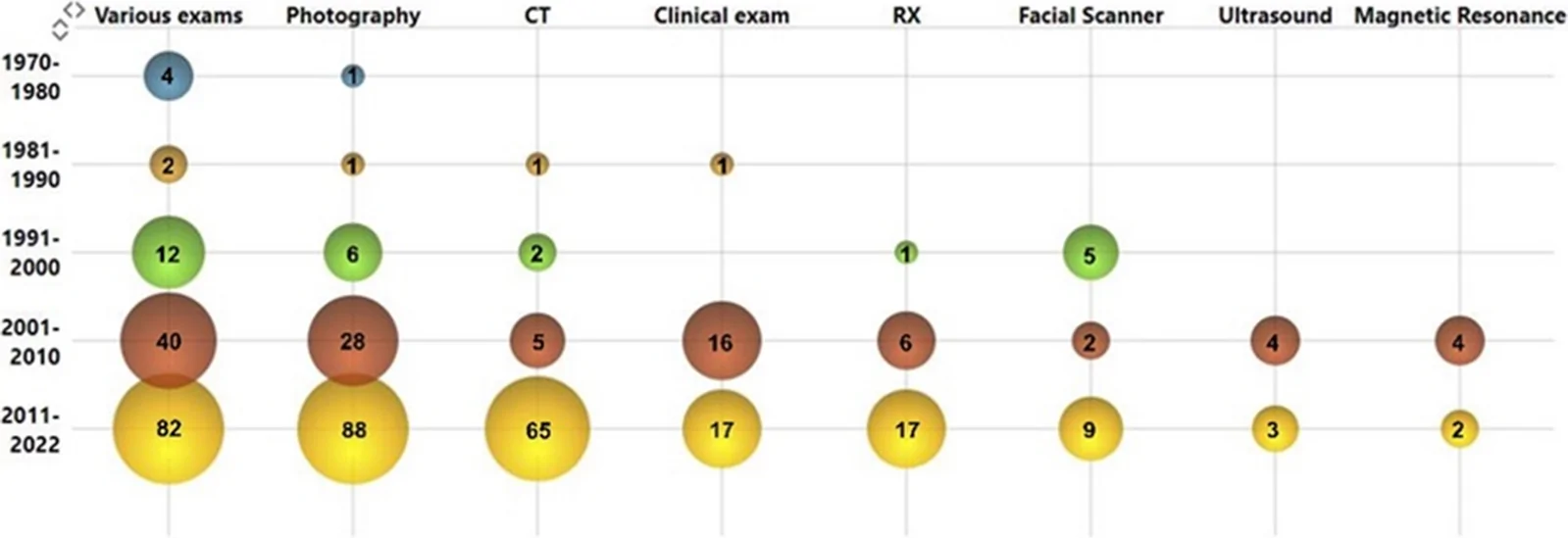
Bubble chart of the type of exams published from 1970 to 2022

All types of applied exams have had an increasing use trend over time since 1970. Only magnetic resonance and ultrasound showed a decrease. The face scanner slightly declined between 2001 and 2010, but its use has grown again in the period between 2011 and 2022. Studies that used more than one type of exam (*n* = 139–32.78%) were more numerous over time. Photography (*n* = 88–20.75%) and computed tomography (*n* = 65–15.33%) were the exams that showed the most significant trends in use in the period between 2011 and 2022 (Fig. 6).

Of the 424 selected studies, 56.37% (*n* = 239) performed a complete face analysis, with 100 achieving more than one analysis, 80 analyzed the entire face metrically, 57 morphologically, and two studies used only volumetric analysis. Studies that analyzed more than one region of the face corresponded to 31.84% (*n* = 135) of the total. From these, the predominance was of studies that metrically analyzed the regions (*n* = 106–25%), followed by those that performed more than one analysis in the regions (*n* = 23–5.42%), and, finally, those that performed morphological analyses (*n* = 6–1.41%).

**Fig. 7:**
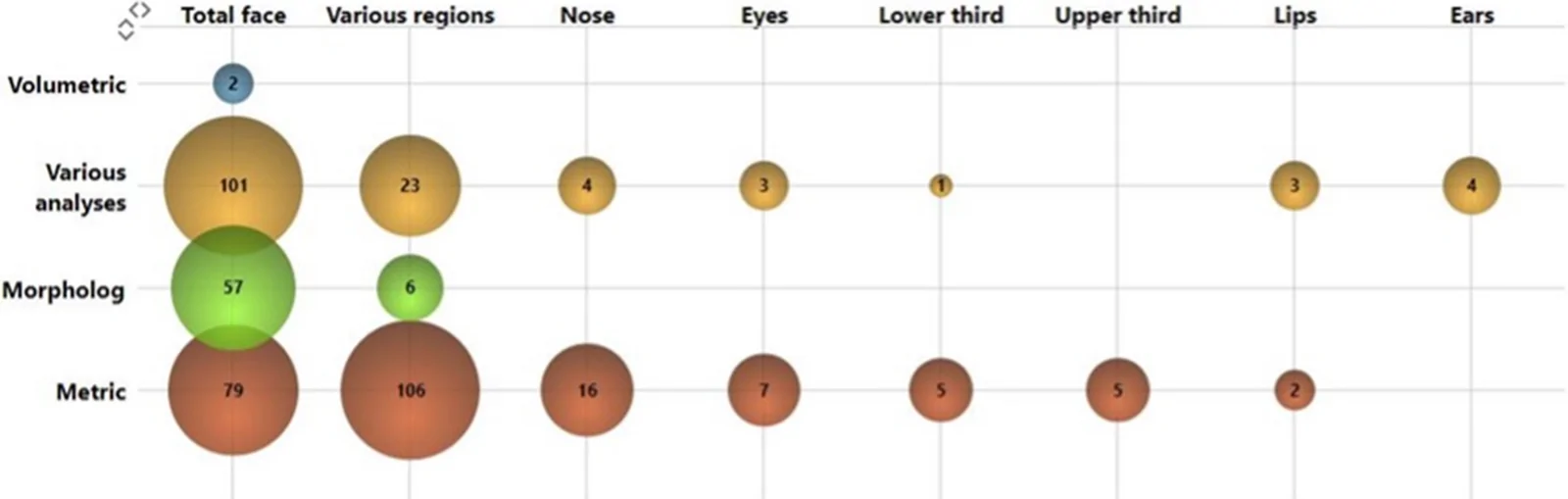
Bubble chart of the type of analysis performed in the face regions that were investigated

The most analyzed isolated region was the nose (*n* = 20–4.72%), with 16 metric analyses and 4 with more than one analysis for this region. The ear was the isolated region that was least analyzed (*n* = 4–0.94%), always analyzed by more than one type of analysis (Fig. 7). Moreover, the most predominant exam of the nose was computed tomography (*n* = 10). The metric analysis was present in 52.12% of the selected studies (*n* = 221), while volumetric analysis was performed in only 0.47% (*n* = 2) (Table 3).

**Table 3:** Correlation between type of analysis, exam, and face regions

Type of exam	Face region	Type of analyses				
		Metric	Morphological	Various analyses	Volumetric	Total
Computed Tomography	Lips	1		1		2
	Lower third	1				1
	Nose	8		2		10
	Total face	14	3	11		28
	Upper third	3				3
	Various regions	20		8		28
Clinical exam	Eyes	2				2
	Total face	12	1	2		15
	Various regions	15		2		17
Facial scanner	Total face	3	2	6	1	12
	Various regions	2	2	1		5
Magnetic resonance images	Ears			1		1
	Eyes			1		1
	Total face	1				1
	Various regions	3				3
Photography	Ears			2		2
	Eyes	4		2		6
	Lips			1		1
	Lower third	3		1		4
	Nose	3				3
	Total face	29	24	21	1	75
	Upper third	1				1
	Various regions	24	3	6		33
Radiography	Lips	1				1
	Nose	4				4
	Total face	4		1		5
	Various regions	13		1		14
Ultrasound	Total face	2		1		3
	Various regions	4				4
Various exams	Ears			1		1
	Eyes	1				1
	Lips			1		1
	Lower third	1				1
	Nose	1		2		3
	Total face	15	27	58		100
	Upper third	1				1
	Various regions	25	1	5		31
	Totals	221	63	138	2	424

Various exams - more than one cited above

**Fig. 8:**
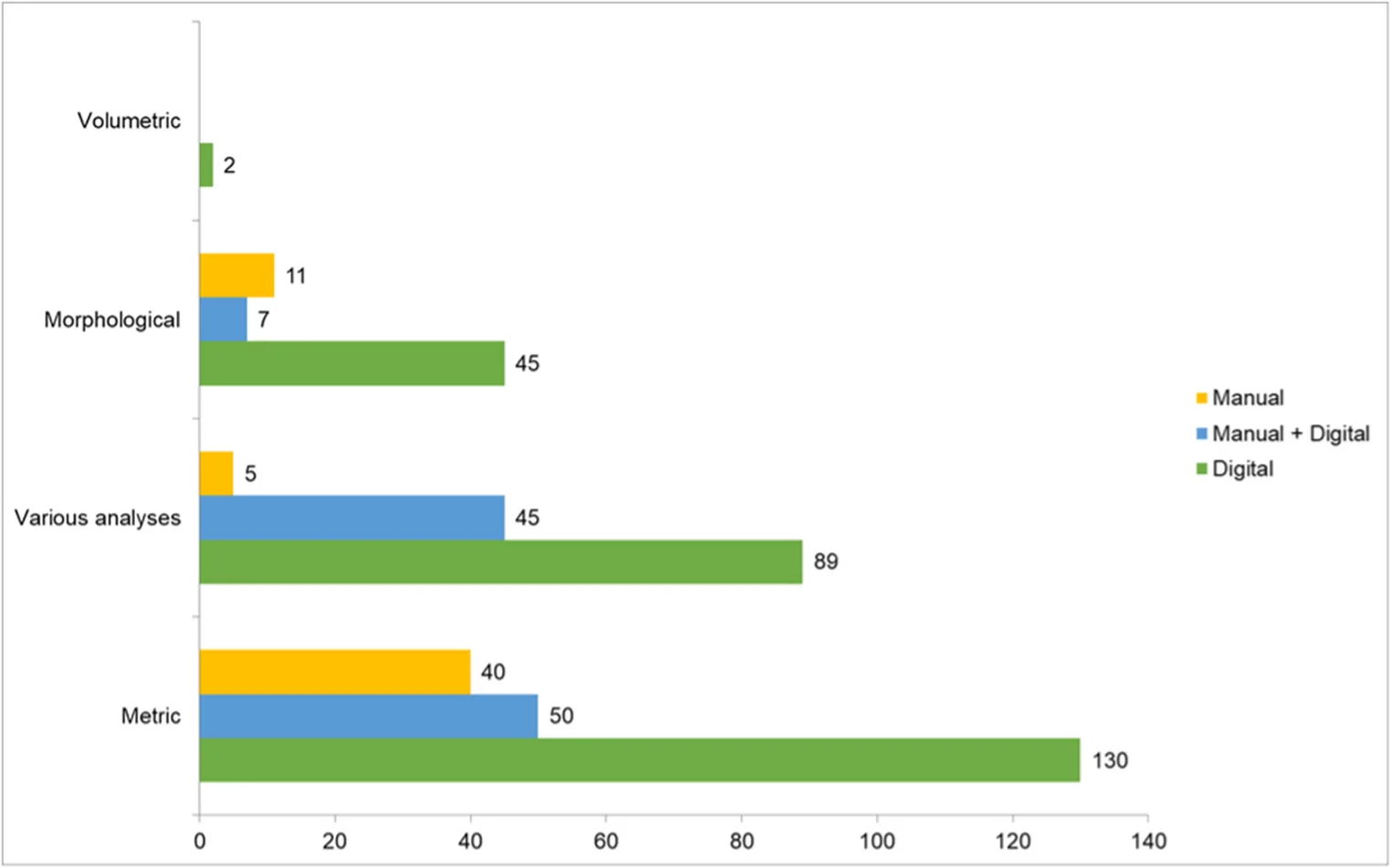
Correlation between type of analysis and method of analysis (manual, digital, and manual-digital together)

Considering the method of analyses (manual/digital), the digital one was used in 266 studies (62.73%), the manual and digital together were present in 103 studies (24.30%), and the manual alone was used in 56 (13.21%). Of the four types of analysis, the only one correlated with one method was volumetric. All studies that analyzed facial regions in the volumetric form used only digital analysis methods. The studies that performed more than one type of analysis, metric and morphological analyses, correlated with the three investigated methods: digital, manual/digital, and manual (Fig. 8).

**Fig. 9:**
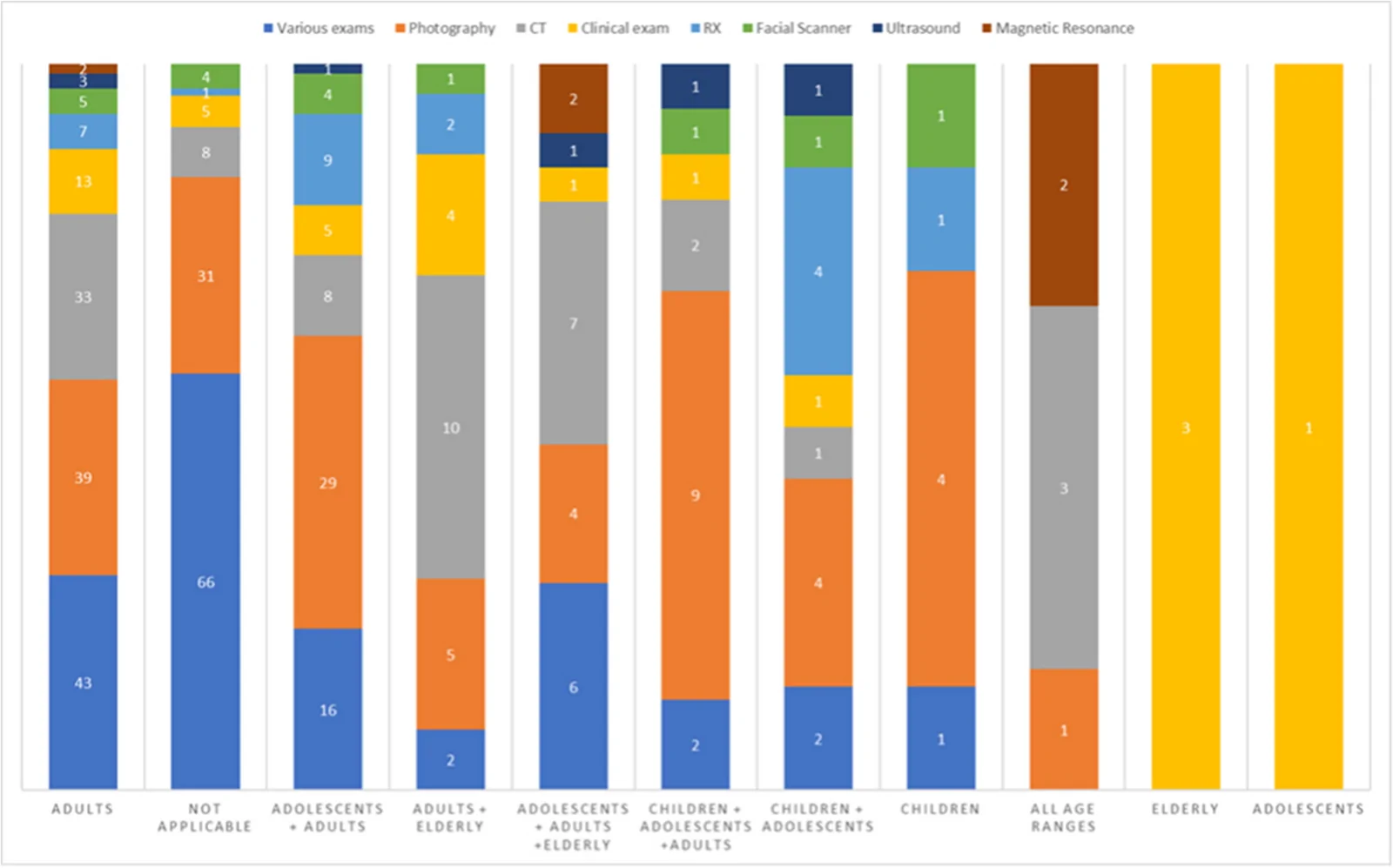
Type of exams performed for human identification considering the different age ranges

There was a predominance of studies with adults (*n* = 145 − 34.20%). However, 115 (27.12%) did not specify the age range analyzed. Children, older people, and adolescents corresponded to fewer studies (*n* = 7–1.65%, *n* = 3–0.71%, *n* = 1–0.23%, respectively), and 6 (*n* = 1.41%) evaluated all age ranges. The remaining studies (*n* = 147–34.67%) searched 2 or 3 age ranges together (adults + older people, adolescents + adults + older people, children + adolescents + adults, children + adolescents). In the studies with adults, all investigated exams were identified, with 43 using more than one type of exam, 39 using photography, and 33 using computed tomography. Only the magnetic resonance exam was not found in the studies that evaluated the age group encompassing adolescents and adults together (Fig. 9).

**Fig. 10:**
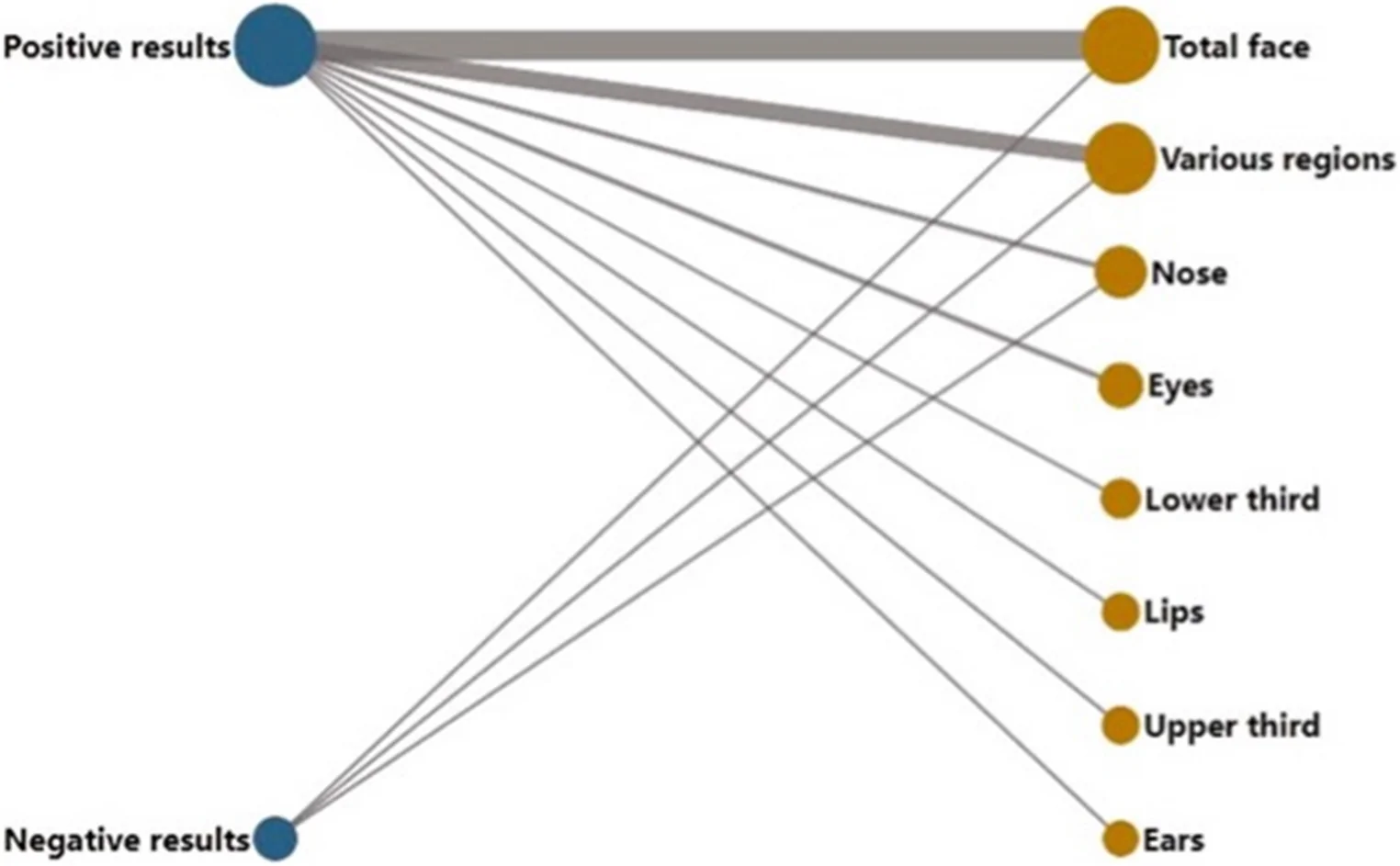
Matrix viewer showing the correlation between the face regions and the results (positive or negative) found by the studies

Of the 424 studies in the present review, 415 (97.88%) presented positive results, and only 9 (2.12%) showed negative results. Of the studies with negative results, 6 analyzed more than one region of the face, 2 analyzed the total face, and only 1 analyzed the nose as an isolated region. The anatomical region called the “middle third”, which encompasses all structures located in the middle third of the face, was not included in any of the studies, although some structures in this region were analyzed separately (Fig. 10).

## Discussion

Human recognition methods through the face are widely described in the literature [2, 11, 14, 15]. Phillips et al. (2018) [13] evaluated the performance of facial identification in groups of humans with different levels of training, experience, talent, and motivation combined with state-of-the-art facial recognition algorithms. They concluded that there is great benefit in the collaborative effort between humans and machines. Another technique widely used in facial recognition, when the face does not present adequate conditions for identification, is the forensic facial approximation [14–17]. Due to its great importance in the world context of forensic sciences [18], a critical review of the world literature seeking the main methods, analyses, and exams for evaluating the face as a human recognition tool is justifiable. The present study makes a critical analysis of these variables mentioned, in addition to other bibliometric data, the central regions of the face studied, and the results obtained about facial recognition, aiming to help researchers and forensic professionals in the knowledge of the main techniques used in human identification through of the face, in this area of primary importance for society.

The forensic use of facial images has significantly increased in recent years. According to the Face Identification Scientific Working Group (FISWG), the analysis of facial features is helpful in intelligence gathering, screening and access control, and forensic identification [19]. The present review found that the most significant % of selected studies (*n* = 273), 64.39%, were published in the period between 2011 and 2022, corroborating the increase in the forensic use of facial images.

Among the bibliometric data evaluated in this review, authors who had several publications equal to or greater than six works were selected, totaling 31 prominent authors whose nationalities represented the five significant continents: two Australian authors, two South Korean, four Japanese, one South African, five Italian, three Germans, one English, one Slovak, four Belgian, one Spanish, two North American and five Brazilian. Recent published systematic reviews of studies that use the face and its regions for human recognition also demonstrated the same diversity of publications’ nationalities [20, 21].

The analysis of the human face, whether by morphological characteristics, metrics, volumetric dimensions, or both, is the object of study in several medical and dental specialities, such as medicine and forensic dentistry, orthodontics, dental prosthesis, dentistry, plastic, and maxillofacial surgery, and, recently, orofacial harmonization. In the present review, the 15 leading journals with four or more published articles were listed: seven journals in forensic sciences, three in craniofacial surgery, one in plastic surgery, one in anatomy, one in orthodontics, and two in multidisciplinary journals. By inserting the use of the face as a means of human recognition as a criterion for inclusion in the studies, logically, a more significant number of studies in medicine and forensic dentistry would be selected, a fact that justifies the more significant number of journals in forensic sciences. In most published studies, the other specialities focus on using the face for diagnoses and treatment plans. Another bibliometric data analyzed in the present review was the article keywords item. It was noticed that, for the most part, the works highlight search words related to the populations of the studies, mainly in terms of age group and sex. Regions studied, types of exams, and areas of science are also presented as the main keywords of the studies, which demonstrated a variety of information about the methods used that enriches relevant information professionals.

The human face is considered the most suitable part of the body for human recognition. Morphological approaches (comparisons of facial shapes), metrics (comparisons of facial size from measurements or indices), and overlays (comparing facial shapes and sizes) are the main scientific techniques for facial assessment [22]. Forensic facial identification using photography as an exam is a common practice to confirm an individual’s identity and their association with criminal events [2, 3, 9, 10]. Associated with all the data mentioned above, the ease of the photographic technique, the low cost of the necessary equipment, the precision of the measurements, and the identification of anthropometric facial points performed indirectly, when compared with the direct technique, make photography an excellent examination method, widespread and accepted. The analysis of the methodologies in the studies selected in the present review confirms photography as the most used type of examination in isolation.

The methods that help in human recognition through the analysis or generation of facial graphics are called facial image methods. These methods include facial approximation or reconstruction, photographic overlay, age regression/progression, construction of facial graphics with data provided by eyewitnesses, facial representation, facial mapping from biological characteristics, analysis of the individual’s D.N.A. (molecular photo fitting), or both, and automated face recognition systems [9, 16, 23–25]. All facial imaging methods use several exams, which provide images of the face or skull of the individual to be identified. Among the different types of exams, the following stand out: photography, visual examination, computed tomography, facial scanning, ultrasound, magnetic resonance imaging, and radiography. Studies that used more than one type of exam as a method were the vast majority in the present review. Almost all the studies included in this review were observational. The comparative method is widely used in this study design; using more than one type of test is well justified.

Computed tomography and radiography exams are widely used to obtain images of the skull (two- or three-dimensional) when there is a need to use facial approximation/reconstruction techniques. Photographs of the dry skull, both two-dimensional and three-dimensional (photogrammetry), are also widely used in this technique. Magnetic resonance imaging and ultrasonography are used in the evaluation of the soft tissues of the face, specifically in studies that perform tissue thickness measurements, which help create population databases that support facial reconstruction techniques. Profile cephalometric radiographs can also be used to measure tissue thickness in different regions of the face. Due to their cost and direct access for human identification, the non-use of such exams justifies the low rate of inclusion of these tests as methods of the studies included in this review.

The analysis of the face, using facial images or through facial reconstructions, is used worldwide in the judicial context and forensic expertise. Most of the studies included in the present review used the total face assessment or even more than one facial region as an effective means of human recognition. The more regions of the face that are analyzed, the greater the probability of this recognition [26, 27]. The methods of analyzing the face by morphology are considered subjective and limited in precision and accuracy, as they depend on the evaluation of expert professionals or people close to the individual to be recognized [28].

Therefore, many facial recognition systems and methods have advanced to metric analysis, with or without descriptive data, showing a high accuracy rate (greater than 95%). Several authors [29–32] confirm that facial metric analysis is a reliable method for human identification since the probability of duplication in several facial measurements is practically null (singularity principle). We saw that in the studies selected in this review, there was a predominance of combined analyses. That is, the face or a specific region was evaluated through two or more types of analyses. Likewise, as previously mentioned about the regions of the beginning, the greater the number of types of analysis, the greater the possibilities of human recognition, which corroborates the result. Metric analysis was predominant in studies that used only one type of analysis, confirming that it is a very effective method for comparisons and facial recognition. Morphological analysis was also found in some studies, mainly due to the greater ease of the comparison method and the relatively low cost of the equipment needed for such analysis. A few studies were observed that used volumetric analysis [25, 33] associated with facial reconstruction techniques and tissue volume variations in different head positions. The need for specific software for this type of analysis, which makes its application in practice brutal, results in a few studies on facial identification.

The selected studies that evaluated isolated regions of the face, for the most part, focused on the nose region, predominantly using metric analysis. By dividing the face into upper, middle, and lower thirds, it was observed that the two anatomical structures most studied in isolation are found in the middle third, according to the results presented in this review: the nose and eyes. However, no studies evaluated both structures, which justifies the non-inclusion of the middle third as a place for their evaluation. In contrast, the upper and lower thirds were cited in some studies as isolated regions studied [34, 35]. Both the nose and the eyes are fundamental structures in the processes of facial reconstruction and characterization of sexual dimorphism; in parallel, several works published in the scientific literature cite specific techniques for such purposes [16, 36–39]. Most of the nose comprises soft tissue and cartilage, both subject to decomposition in bodies that need identification. Therefore, research on the relationship between the nasal opening, the hard tissue part of the nose, and the soft tissue is carried out in different populations to facilitate facial reconstruction [39]. In the period between 2011 and 2021, it was observed that such studies focused on Asian populations since there is a lack of data to compose information banks related to the populations of this continent [40, 41]. Computed tomography exams sufficiently reproduce the shape of the actual skull, allowing complete visualization of hard and soft tissue; therefore, it is the type of exam most used in such studies.

With the broad development of digital technology, scientific research is constantly seeking to bring to society all the possible benefits arising from technological innovations. Digital exams and analysis methods ensure greater accuracy and precision in the results and provide additional information not accessible in analogue methods [39, 42–44]. All this technological growth makes the cost increasingly accessible, facilitating the use of digital methodologies. Over half of the studies included in this review used digital analysis methods, confirming the search for more assertive, precise, and accurate methodologies. To a lesser extent, due to the low resources allocated to research in several countries, some studies used manual and digital analysis methods. Only manual methods are increasingly scarce since they bring possibilities of errors, subjective evaluations, and less precision in the results of scientific works, a fact proven in this research, having been the method least applied in the selected studies.

The population of the studies selected in the present review was predominantly composed of adult individuals (20 to 59 years of age, according to the World Health Organization, WHO), as well as more than one age group included in the same study (*n* = 145 adults and *n* = 147 more than one age group), even in studies with individuals of more than one age group, adult individuals predominated among the different groups. Some factors may have contributed to this result: Greater acceptance of adults as volunteers in scientific research, a more significant number of facial image exams performed on adults, the age group with fewer morphological, metric, and volumetric alterations in different facial structures, and even the age group more involved in accidental and criminal events. Individuals belonging to the groups of children, adolescents, and elderly were present, in isolation, in only 11 studies in total, a result explained for reasons opposite to those presented for the group of adults, mentioned above.

A significant part of the studies (*n* = 115) did not specify the age groups of the population studied. In these cases, the studies evaluated unknown skulls, random images captured by surveillance cameras, or radiological exam files. Regarding the types of exams found in the selected studies, only those that included the adult age group were found to have all types of exams performed. The ease of monitoring this group of individuals is probably one more reason for this result.

The majority of the analyzed literature reported favorable outcomes regarding facial recognition and identification, with only nine studies (2.12%) presenting negative results. It is important to clarify that the labels ‘positive’ and ‘negative’ in this bibliometric analysis reflect the qualitative effectiveness and promising trend of these methods, rather than a quantitative success rate. However, this low percentage of negative results should be interpreted with caution, as it likely reflects a publication bias toward successful identification methods rather than the absolute infallibility of the techniques. Within this trend, the prominent research interest in the nasal region underscores a move toward refining facial approximation, suggesting that future studies should further explore the correlation between soft and hard tissues to enhance reconstruction accuracy.

The predominance of digital methods in this review (62.73%) highlights a significant shift in forensic facial analysis, where automated face recognition systems (AFRS) are progressively reducing the traditional reliance on manual metric analysis. This transition reflects a critical human-machine collaboration: while Artificial Intelligence (AI)-driven tools enhance objectivity and processing speed, the forensic expert’s role remains indispensable for interpreting complex cases and validating results to meet the rigorous standards of legal evidence.

Despite these contributions, some limitations must be acknowledged. First, the image samples used in the reviewed studies often originate from single-centre databases, which may restrict the generalizability of findings across different cone-beam computed tomography (CBCT) devices and populations. The inherent complexity of anatomical variations and differing bone densities also remains a challenge for the precision of automatic segmentations. To overcome these boundaries, future research should prioritize reporting unsuccessful cases or borderline accuracies, as these provide essential data for understanding the limitations of both manual and digital methods. Furthermore, involving multicenter databases and standardized image acquisition protocols is essential to consolidate the robustness and transparency of AI tools in forensic practice.

## Conclusion

In the period between 2011 and 2022, there has been a growing interest in human facial recognition within the scientific community, establishing facial analysis methods as an increasingly common reality in forensic practices. While photography remains the most utilized exam, 3D methods such as computed tomography and facial scanning are becoming more prominent. Bibliometric data reveal that analysis of the entire face is the most frequent approach for human recognition; however, when localized regions are evaluated, the nose stands out as the most studied area. Furthermore, digital methods and the use of anthropometric points prevail as the leading techniques in facial comparisons.

In conclusion, the evolution of these techniques points toward the increasing integration of AI into diagnostic imaging protocols, emerging as a valuable tool to optimize forensic and radiological workflows. The trends identified suggest that 3D volumetric and anthropometric analyses performed by algorithms are gaining ground due to their potential for superior precision in evaluating complex structures compared to conventional 2D methods. Thus, the role of AI is being established as an essential support for future practices, enabling faster and more accurate quantitative analyses, which directly contribute to more assertive human identification and specialized forensic planning.

## Supplementary Information

Below is the link to the electronic supplementary material.

Supplementary Material 1

## Data Availability

The datasets generated and/or analyzed during the current study are available from the corresponding author upon reasonable request.

## Abbreviations

PM: Post-mortem
INTERPOL: International criminal police organization
CT: Computer tomography
PRISMA: Preferred reporting items for systematic reviews and meta-analyses
OSF: Open science framework
FISWG: Face identification scientific working group
WHO: World health organization
AFRS: Automated face recognition systems
AI: Artificial intelligence
CBCT: Cone beam computed tomography

## Declaration

### Submission declaration

This work has not been published previously, it is not under consideration for publication elsewhere. Its publication is approved by all authors and tacitly or explicitly by the responsible authorities where the work was carried out, and that, if accepted, it will not be published elsewhere. The abstract of this study was published in the annals and presented as a poster at the 39th annual meeting of the Brazilian Society of Dental Research in September 2022.
